# Human Ribosomal Proteins RPeL27, RPeL43, and RPeL41 Are Upregulated in Nasopharyngeal Carcinoma Cell Lines

**DOI:** 10.1155/2016/5179594

**Published:** 2016-11-28

**Authors:** Edmund Ui-Hang Sim, Stella Li-Li Chan, Kher-Lee Ng, Choon-Weng Lee, Kumaran Narayanan

**Affiliations:** ^1^Department of Molecular Biology, Universiti Malaysia Sarawak, 94300 Kota Samarahan, Malaysia; ^2^Institute of Biological Sciences, University of Malaya, 50603 Kuala Lumpur, Malaysia; ^3^School of Science, Monash University, Bandar Sunway, 46150 Selangor, Malaysia; ^4^Department of Genetics and Genomics Sciences, Mount Sinai School of Medicine, New York, NY 10029, USA

## Abstract

Apart from their canonical role in ribosome biogenesis, there is increasing evidence of ribosomal protein genes' involvement in various cancers. A previous study by us revealed significant differential expression of three ribosomal protein genes (*RPeL27*,* RPeL41*, and* RPeL43*) between cell lines derived from tumor and normal nasopharyngeal epithelium. However, the results therein were based on a semiquantitative assay, thus preliminary in nature. Herein, we provide findings of a deeper analysis of these three genes in the context to nasopharyngeal carcinoma (NPC) tumorigenesis. Their expression patterns were analyzed in a more quantitative manner at transcript level. Their protein expression levels were also investigated. We showed results that are contrary to previous report. Rather than downregulation, these genes were significantly overexpressed in NPC cell lines compared to normal control at both transcript and protein levels. Nevertheless, their association with NPC has been established. Immunoprecipitation pulldown assays indicate the plausible interaction of either RPeL27 or RPeL43 with POTEE/TUBA1A and ACTB/ACTBL2 complexes. In addition, RPeL43 is shown to bind with MRAS and EIF2S1 proteins in a NPC cell line (HK1). Our findings support* RPeL27*,* RPeL41*, and* RPeL43 *as potential markers of NPC and provide insights into the interaction targets of RPeL27 and RPeL43 proteins.

## 1. Introduction

Ribosomal proteins (RPs) are primarily known for their functions in ribosome biogenesis and play a central role in translational processes. In fact, the highly coordinated processes of ribosome biogenesis are also tightly connected to events of cellular growth and development. Dysregulation in these processes could relate to occurrence of diseases that include cancers. It is also an established fact that the phenotypic effects of RP genes extend beyond their canonical ribosomal involvement into extraribosomal functions such as DNA replication, transcription, DNA repair, DNA splicing and modification, and apoptosis [1]. In particular, differential expression of ribosomal proteins (RPs) has also been related to cancers [2, 3]. Recently all ribosomal protein genes have been accorded new nomenclature [4], and this is used in this paper we provide, but the old names are provided at their first mention in the text.

Nasopharyngeal carcinoma (NPC), a malignancy arising from epithelial cells of the nasopharynx, is a cancer that has been extensively studied with respect to genetic susceptibility and involvement. Early evidence of RP genes involvement in NPC was limited to RPeS26 (RPS26), RPeS27 (RPS27), RPuS19 (RPS15), RPeL27 (RPL27), RPeL43 (RPL37a), and RPeL41 (RPL41) [5–7]. Albeit providing information on NPC-associated RP genes, these preliminary findings are largely speculative due to analysis that are semiquantitative in nature and/or confined to assessment at transcript level. Indeed, inconsistent results of RPeS26 and RPeS27 in another study [8] nullified the verity of these two RP genes as NPC-associated factors. The case of RPuS19, although identified from a large list of differential expressed genes (via microarray assay) between NPC and noncancerous nasopharyngeal tissue samples [6], was not subsequently selected for validation via conventional or quantitative RT-PCR analysis. Its upregulation in NPC samples was also not evaluated at protein level. The three RP genes, RPeL27, RPeL43, and RPeL41, were identified to be associated with NPC from a study that employed semiquantitative RT-PCR assay of all RP genes encoding products for the large ribosome subunit [7]. Their underexpression in NPC cell lines compared to normal epithelial cell line remains to be reconfirmed using quantitative RT-PCR and/or Western Blot analysis. Without definitive results of expression pattern and functional implications, the notion of NPC-associated RP factors continues to be provisional and elusive.

Molecular pathways or signaling events pertaining to carcinogenesis of NPC, hitherto, cannot categorically include the involvement of RPs. Therefore, to establish RPs as among the factors associated with NPC tumorigenesis, a reevaluation of their expression patterns using more quantitative assay is warranted. This paper reports the reanalysis of expressed transcript level using quantitative RT-PCR strategy of RPeL27, RPeL43, and RPeL41 in NPC cell lines in comparison to normal cell line derived from noncancerous nasopharyngeal epithelium. The upregulated trend of two of these three genes was further substantiated at the protein level, thus validating their association with NPC. Further study on protein-protein interaction reveals plausible binding of two of these three RPs with protein complexes of the cell cytoskeleton, while one of the RPs was shown to possibly interact with two types of oncoprotein.

## 2. Materials and Methods

### 2.1. Cell Culture

Nasopharyngeal carcinoma cell lines used were SUNE1, HONE1, HK1, and TW01, and nonmalignant nasopharyngeal epithelial cell line was NP69 [9]. Original source and consent for use of these cell lines were provided by Tsao et al., University of Hong Kong (Hong Kong). The NP69 cells were cultured in defined keratinocyte-serum-free medium (Invitrogen) supplemented with 5% fetal bovine serum (Gibco), 100 U/mL penicillin-streptomycin (Gibco), and 0.2 ng/mL recombinant epidermal growth factor (Gibco), while SUNE1, HONE1, HK1, and TW01 cells were grown in RPMI-1640 (Invitrogen) supplemented with 10% FBS (Gibco) and 100 U/mL penicillin-streptomycin (Gibco). All cells were maintained at 37°C with 5% carbon dioxide.

### 2.2. Quantitative Reverse Transcription-Polymerase Chain Reaction (qRT-PCR)

Total RNA was extracted with TRIzol reagent (Invitrogen) according to the manufacturer's protocol. First-strand cDNA was synthesized using QuantiTect Reverse Transcription Kit (Qiagen) by standard method. Real-time quantitative reverse transcription-PCR (qRT-PCR) was performed in three technical replicates with two biological replicates using QuantiFast Probe (Qiagen) on a Rotor-Gene 6000 Rotary Analyzer (Qiagen) and monitored with Rotor-Gene 6000 software version 2.3.3 (Qiagen). The primer-probe pairs used targeted RPeL27, RPeL41, and RPeL43 genes. Beta actin (ACTB) was used as endogenous control for gene expression normalization. Nontemplate reactions were included as negative controls. The fold changes were calculated by using 2^−ΔΔCt^ method.

### 2.3. Western Blotting

Whole cell protein lysates were prepared using RIPA lysis buffer containing protease inhibitor (Roche Applied Sciences). Proteins were separated on 12.5% SDS-polyacrylamide gel electrophoresis and transferred onto nitrocellulose membrane (Milipore). After blocking with bovine serum albumin, the membranes were blotted with the primary antibodies for RPeL27 or RPeL43 (Abcam), followed by secondary anti-donkey, rabbit secondary antibody (Abcam). ACTB was used as protein loading control. Analysis was not performed for RPeL41 due to lack of commercially available primary antibody. Protein bands were visualized using enhanced chemiluminescent (eCL) substrate (Promega). Images were captured using ImageQuant TL instrument (GE Healthcare) and band intensity was read using ImageQuant TL software.

### 2.4. Immunoprecipitation Pulldown Assay

Whole cell protein lysate extracted from a NPC cell line (HK1) was incubated with RPeL27 or RPeL43 primary antibody immobilized onto Dynabeads Protein G (Life Technologies) for 10 minutes with rotation at room temperature. The beads were vortexed for 30 seconds, and the vial was placed on DynaMag Magnet (Life Technologies) to separate them from the solution. Following removal of supernatant, sample buffer was added to the vial and this mixture was boiled for 10 minutes. The resulting immunoprecipitates were resolved on SDS-PAGE and stained with Coomassie Blue (Bio-Rad). Protein bands of interest were excised and sent to a service provider for protein identification. Identification analysis was via the Electrospray Ionization-Quadrupole-Time-of-Flight (ESI-QUAD-TOF) method using the Agilent 1260 Infinity HPLC system coupled to Agilent 6540 mass spectrometer (Agilent) and the Matrix Assisted Laser Desorption/Ionization Time-of-Flight (MALDI-TOF) method using the 5800 Protein Analyzer (AB Sciex). Identities of proteins were extracted using Mascot software (Matrix Science) on Swiss-Prot database. The search parameters utilized trypsin as the proteolytic enzyme with one missed cleavage permitted and the carbamidomethylation of cysteines and oxidation of methionines as variable modifications. Mass tolerance was 50 ppm and 0.4 Da for precursor and fragment ions, respectively.

## 3. Results

### 3.1. Overexpression of RPeL27, RPeL43, and RPeL41 in NPC Cells

**Figure 1:**
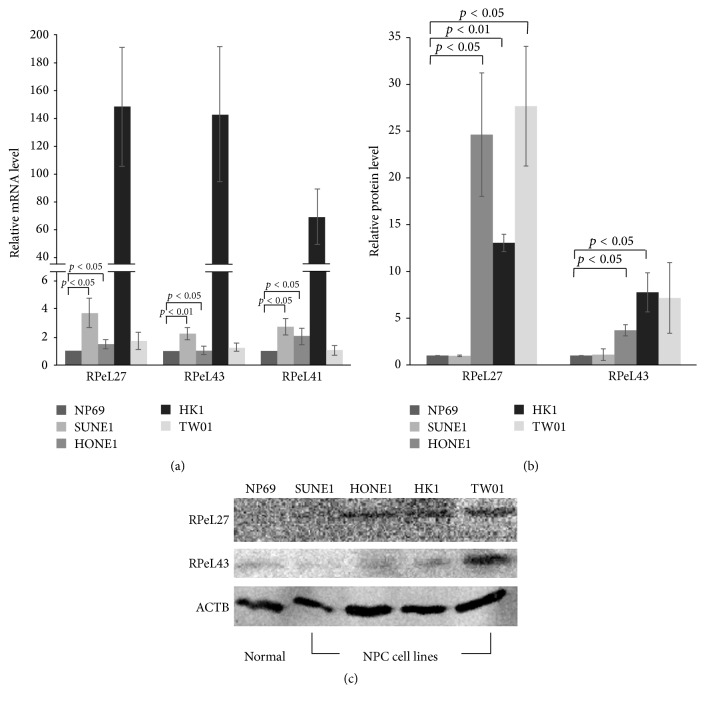
RPeL27, RPeL43, and RPeL41 were overexpressed in transcript and protein level. (a) Bar graph with error bars of quantitative RT-PCR assays measuring mRNA of all three RP genes in the five cell lines studied. ((b) and (c)) Western blotting analysis results of protein levels of RPeL27, RPeL43, and RPeL41 in five cell lines studied. Beta actin (ACTB) protein (Accession NM 001101 was used as reference and loading control where values from RPs were normalized with).

Based on our quantitative RT-qPCR results, all three RP genes (RPeL27, RPeL43, and RPeL41) show overexpression in all NPC cell lines compared to normal nasopharyngeal epithelial cells, while our Western Blot analysis reveals upregulated protein level of RPeL27 and RPeL43 in NPC cell lines relative to a normal control (Figure 1). Transcript levels of RPeL27 in SUNE1 and HONE1 cell lines were 3.73- and 1.51-fold higher (*p* = 0.044 and 0.011) than in the normal nasopharyngeal epithelial cell line, NP69; and RPeL43 mRNA in SUNE1 and HONE1 cell lines was 2.23- (*p* = 0.009) and 1.04-fold (*p* = 0.012) higher, respectively, compared to NP69 (Figure 1(a)). This overexpression pattern is also seen in RPeL41 with 2.74-fold in SUNE1 (*p* = 0.013) and 2.05-fold in HONE1 (*p* = 0.028). However, the fold difference of the three RP genes' mRNA in HK1 and TW01 cell lines is not statistically significant, albeit large differential expression of all three RP genes between HK1 and NP69 cell lines (Figure 1(a)). In terms of transcript level, RPeL27 shows the highest expression in all NPC cell lines studied (Figure 1(a)).

Western Blot analysis shows upregulated protein levels of RPeL27 and RPeL43 in the NPC cell lines of HONE1, HK1, and TW01 compared to NP69 (Figures 1(b) and 1(c)). RPeL27 is 24.62 (*p* < 0.05), 13.05 (*p* < 0.01), and 27.67 (*p* < 0.05) in HONE1, HK1, and TW01 cell lines, respectively, compared to NP69 cell line. Meanwhile, RPL37a protein level was 3.71 and 7.76 (*p* < 0.05) in HONE1 and HK1 cell line, respectively. In both protein and transcript levels, RPL27 showed overall higher expression across the NPC cell lines compared to RPL37a. ACTB (Accession NM 001101) was used as reference gene and protein loading control to normalize the expression.

### 3.2. Proteins Interacting with RPeL27 and RPeL43

**Figure 2:**
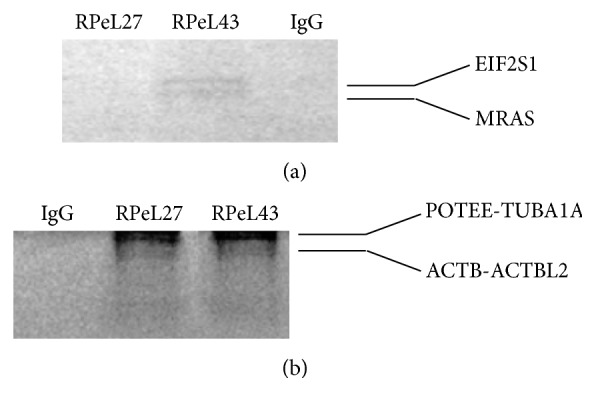
Protein-protein analysis using coimmunoprecipitation assay. (a) EIF2S1 and MRAS proteins were pulled down along with RPeL43. (b) POTEE protein in complex with TUBA1A is the heavier protein complex that coimmunoprecipitates with both RPeL27 and RPeL43, respectively, while the lighter complex was ACTB and ACTBL2 protein. IgG is negative control for coimmunoprecipitation assay where antibody is not included to check for nonspecific binding of other proteins with protein G on beads.

Based on ESI-QUAD-TOF mass spectrum analysis, we identified Muscle Rat Sarcoma Homolog (MRAS) and Eukaryotic Initiation Factor 2 Subunit 1 (EIF2S1) proteins as coimmunoprecipitates of the RPeL43 protein (Table 1). The predicted size from ESI-QUAD-TOF result matches the relative size of the protein band in SDS-PAGE (25–35 kDa) (Figure 2(a)). Heavier proteins in our SDS-PAGE assays that coimmunoprecipitate with each of the RPeL27 and RPeL43 proteins (Figure 2(b)) were later identified as POTE Ankyrin Family Member E- tubulin alpha 1A (POTEE-TUBA1A) and beta actin-beta actin-like 2 (ACTB-ACTBL2) complexes via the MALDI-TOF analysis (Table 2). Both RPeL27 and RPeL43 pulled down the same complexes as they have similar weight in SDS-PAGE. Heavy chain and light chain of the two primary antibodies RPeL27 and RPeL43 were detected but faint (not shown).

## 4. Discussion

Our results on the significantly upregulated levels of RPeL27, RPeL43, and RPeL41 transcripts in NPC cell lines compared to cultured normal NP cells warrant further discussion. While this expression phenomenon reinforced the fact that they are likely candidates of NPC-associated RP factors, their expression behaviors contradict our previous report [7] of downregulation in NPC cells. Such discrepancy is difficult to explain, but, needless to say, our current findings are more definitive due to the greater accuracy of quantitative RT-PCR over semiquantitative strategy. Our current results also reveal the upregulated trend of RPeL27 and RPeL43 protein levels in NPC cells, thus substantiating and validating the outcomes of transcript assessment. On this note, our previous report that evaluated the end-point transcript level of these RP genes in NPC scenario [7] will now have to be taken with caution.

The overexpression trends of these three RP genes are not uncommon in cancers. For instance,* RPeL27* is upregulated in cells/tissues of sulforaphane treated-breast cancers [10], gastric tubular adenoma and carcinoma [11], hepatocellular carcinoma (HCC) [12], and metastatic liver lesions [13], while* RPeL43* is upregulated in cases of astrocytomas [14], head and neck squamous cell carcinoma [15, 16], and hepatitis B-associated HCC [17]. The RPeL41 gene has been found to be overexpressed in neoplastic lung fibroblast cells [18]. Our findings are the first to show their overexpression in a variety of cell lines derived from NPC tissues and subsequently verified their status as plausible NPC-associated genetic factors.

In terms of cell types, significantly higher transcript level of the three RP genes is observed for cell lines derived from the more common type of NPC tissues (Type 2a) comprising nonkeratinized poorly differentiated cells (SUNE1 and HONE1). Despite prevailing overexpression, statistical significance is not apparent in the case of cell lines from well-differentiated NPC cells of either keratinized (TW01) or nonkeratinized (HK1) characteristics. Protein levels of RPeL27 are significantly higher in the three types of cell lines, namely, the nonkeratinized poorly differentiated (SUNE1/Type 2a-NPC), nonkeratinized well-differentiated (HK1/Type 2b-NPC), and keratinized well-differentiated (TW01/Type 1-NPC) cell types. Higher levels of RPeL43 proteins are statistically significant in all cell types except for keratinized well-differentiated ones (TWO1). Taken together, it is clear that this subset of RP genes is most consistently overexpressed in cells from Type 2a-NPC tissues, hence conferring them possible biomarkers for detection and/or progression of this type of NPC. Our findings here provide the first empirical evidence to support this conclusion. Nevertheless, we have yet to further investigate all these observations or prove these inferences in NPC tissues.

One of the proteins we found interacting with RPeL43 is EIF2S1. This association is not a surprise bearing in mind that EIF2S1 primarily functions as a translational initiation factor, and its phosphorylation has been known to inhibit protein synthesis [19]. In leukemia cells, the phosphorylation of EIF2S1 also inhibits the activities of nicotinamide phosphoribosyltransferase (NAMPT), a factor known to regulate cancer cell metabolism [20]. Exactly how the interaction among RPeL43, EIF2S1, and NAMPT attributes to dysfunctional or normal growth and proliferation of nasopharyngeal epithelial cells is unclear and can only be elucidated via further functional studies. However, our limited findings here do suggest a probable role of RPeL43 in cell growth regulation when in complex with EIF2S1.

Another protein identified to be in complex with RPeL43 is MRAS. As activator of other protooncogenes such as Rapidly Accelerated Fibrosarcoma (RAF) kinases [21], Phosphoinositide-3-Kinase (P13K) [22], Afadin (AF6) [23], and Guanine Nucleotide Exchange Factors (GEFs) [24], MRAS also triggers Mitogen-Activated Protein Kinases/Extracellular Signal-Regulated Kinases- (MAPK/ERK-) independent gene expression in breast cancer cells [25]. Our studies are the first to identify possible association between an RP and the MRAS protein in the NPC context. Other Ras family members previously implied in NPC-related tumorigenesis are V-Ki-ras2 Kirsten Rat Sarcoma Viral Oncogene Homolog (KRAS) and Transforming Protein p21 (HRAS) [26].

For the heavier molecules (POTEE-TUBA1A and ACTB-ACTBL2 complexes) that coimmunoprecipitated with RPeL27 or RPeL43, precise mode of interaction (one-to-one binding) cannot be discerned via our analysis. Each RP's interaction with either one of the complexes could be due to protein aggregation in the assay or actual tertiary structures in the natural state. Under normal circumstances, POTEE (or POTE-2*γ*) colocalizes with actin beneath the cell membrane [27] but has been known to translocate from cytoplasm to nucleoli in response to conditions of malignancy [28]. Whether and how RPeL27 or RPeL43 is involved in this event during NPC tumorigenesis remains to be studied. Despite expression in normal tissues of the prostate, ovary, testis, and placenta (hence the acronym POTE), transcripts of POTEE gene are also detected in tissues of prostate, breast, lung, colon, and ovarian cancers [29]. In the current study, we reveal the presence of POTEE proteins in NPC cells, albeit in association with RPeL27-TUBA1A or RPeL43-TUBA1A complexes. Among the tubulin superfamily, only beta-tubulin family member, specifically the beta 2-tubulin chain (TBB2), is possibly associated with NPC in that its protein level is downregulated in an Epstein-Barr virus-associated NPC cell line (C666-1) [30]. On the other hand, none of the alpha-tubulin family members has been linked to NPC before this study, despite their association with prostate cancer [31] and neuroblastoma [32]. The tubulin alpha-1A (TUBA1A) has only been linked to non-small cell lung carcinomas [33]. Our findings herein are the first to suggest its involvement in NPC tumorigenesis albeit of mechanism yet to be discovered.

In the case of RPeL27-ACTB-ACTBL2 or RPeL43-ACTB-ACTBL2 complexes, little is known about the roles of either complex in NPC or any other cancer situations. Indeed, the association of ACTB with a wide range of cancer types (liver, renal, colorectal, gastric, pancreatic, esophageal, lung, breast, prostate, and ovarian cancers and leukemia, melanoma, and lymphoma) has been explained [34] and that of ACTBL2 in some hepatocellular carcinoma has been reported [35]. However, the combined effects of ACTB, ACTBL2, and either RPeL27 or RPeL43 in malignant transformation of human cells are unclear. Taking together the findings from our co-IP assays, we could perhaps suspect an RPeL27- and RPeL43-aided involvement of the MAPK/ERK pathway in malignancy of the nasopharynx since factors such as MRAS and members of actin and tubulin families have been identified. This hunch can be among the bases for deeper studies into RP-mediated mechanism in molecular pathogenesis of NPC.

## 5. Conclusion

This study verified* RPeL27*,* RPeL41*, and* RPeL43* as NPC-associated ribosomal protein genes as far as cell line system is concerned. We provide preliminary knowledge on the development of novel markers for early diagnosis and/or prognosis of the nasopharyngeal cancer disease. In addition, we provide early evidence suggesting possible roles of RPeL27 and RPeL43 in the MAPK/ERK pathway.

## Figures and Tables

**Table 1:** Identification of proteins immunoprecipitated with RPeL27 or RPeL43 in NPC by ESI-QUAD-TOF MS. MW indicates molecular weight. Accession number refers to SWISS-PROT protein database. Matches are the count of MS/MS spectra that have been matched to peptides from this protein. Ion-score or peptide score is a probabilistic score indicating the goodness-of-fit between the observed MS/MS spectrum and the theoretical spectrum of the proposed peptide. A higher score indicates a higher probability of a nonspurious match. Significant threshold or *p* value is used to determine statistical significance of matches between the observed and theoretical MS/MS spectra. Sequence coverage is sequence of amino acids that has been inferred to be present based on mass spectral evidence.

Protein name	MW (kDa)	Accession number	Matches	Ion-score/*p* value	Sequence coverage
EIF2S1	36089	P05198	10	31/18	16%
MRAS	23831	O14807	4	26/51	14%

**Table 2:** Identification of proteins immunoprecipitated with RPeL27 or RPeL43 in NPC by ESI-QUAD-TOF MS. MW indicates molecular weight. Accession number refers to SWISS-PROT protein database. Matches are the count of MS/MS spectra that have been matched to peptides from this protein. Ion-score or peptide score is a probabilistic score indicating the goodness-of-fit between the observed MS/MS spectrum and the theoretical spectrum of the proposed peptide. A higher score indicates a higher probability of a nonspurious match. Significant threshold or *p* value is used to determine statistical significance of matches between the observed and theoretical MS/MS spectra. Sequence coverage is sequence of amino acids that has been inferred to be present based on mass spectral evidence.

Protein name	MW (kDa)	Accession number	Matches	Ion-score/*p* value	Sequence coverage
POTEE	121286	Q6S8J3	2	94/<0.05	2%
TUBA1A	50104	F8VQQ4	2	33/<0.05	7%
ACTB	41710	P60709	3	73/<0.05	12%
ACTBL2	41976	Q562R1	2	50/<0.05	9%
